# Killing Effect of Ad5/F35-APE1 siRNA Recombinant Adenovirus in Combination with Hematoporphrphyrin Derivative-Mediated Photodynamic Therapy on Human Nonsmall Cell Lung Cancer

**DOI:** 10.1155/2013/957913

**Published:** 2012-12-27

**Authors:** Lei Xia, Wei Guan, Dong Wang, Yun-Song Zhang, Lin-Li Zeng, Zeng-Peng Li, Ge Wang, Zhen-Zhou Yang

**Affiliations:** ^1^Cancer Center, Research Institute of Surgery, Daping Hospital, Third Military Medical University, Chongqing 400042, China; ^2^Department of Thoracic Surgery, Research Institute of Surgery, Daping Hospital, Third Military Medical University, Chongqing 400042, China; ^3^Department of Pathology, Research Institute of Surgery, Daping Hospital, Third Military Medical University, Chongqing 400042, China

## Abstract

The main goal of this work is to investigate the killing effects and molecular mechanism of photodynamic therapy (PDT) mediated by the Ad5/F35-APE1 siRNA recombinant adenovirus in combination with a hematoporphrphyrin derivative (HpD) in the A549 human lung adenocarcinoma cell line in vitro to provide a theoretical reference for treating lung cancer by HpD-PDT. By using the technologies of MTT, flow cytometry, ELISA, and western blot, we observed that the proliferation inhibition and apoptosis of the A549 cells were significantly higher than the control group (*P* < 0.05) after HpD-PDT was performed. The inhibitory efficiency is dependent on the HpD concentration and laser intensity dose. The inhibitory effect on the proliferation of A549 cells of Ad5/F35-APE1 siRNA is more significant after combining with PDT, as indicated by a significant elevation of the intracellular ROS level and the expression of inflammatory factors (*P* < 0.05). The HpD-PDT-induced expression of the APE1 protein reached the peak after 24 h in A549 cells. The inhibition of APE1 expression in A549 cells was most significant after 48 hours of infection by Ad5/F35-APE1 siRNA recombinant adenovirus (10 MOI). In conclusion, the Ad5/F35-APE1 siRNA recombinant adenovirus could efficiently inhibit the HpD-PDT-induced APE1 expression hence could significantly enhance the killing effect of HpD-PDT in lung cancer cells.

## 1. Introduction

Lung cancer is one of the most common malignant cancers in humans and has become the leading cause of death among malignant cancers. Lung cancer's incidence and mortality rate gradually increase each year. Despite the rapid progress of surgery, radiotherapy, chemotherapy, and biotherapy, the long-term survival rate of patients with lung cancer remains poor, and new therapeutic strategies are urgently needed [1, 2]. Photodynamic therapy (PDT) mediated by hematoporphrphyrin derivative (HpD) is a minimally invasive tumor treatment in which tumor cells selectively absorb photosensitizers and are killed by a photochemical reaction that is generated from photosensitizers activated by laser irradiation at a specific wavelength. This therapy plays an important role in lung cancer treatment [3, 4]. Under aerobic conditions, PDT can directly impair tumor cells by producing reactive oxygen species (ROS) through the irradiation by photosensitizers of the target cells using an adequate light wavelength [5, 6]. ROS can damage capillaries, leading to reduced blood flow to tumor tissues and the generation of multiple inflammatory factors that induce apoptosis or necrosis. Lung cancer cells can express apurinic/apyrimidinic endonuclease (APE1) [7], which is a vital protective gene that shields cells from oxidative stress- and hypoxia-induced apoptosis and necrosis, and this gene has the dual functions of DNA damage repair and oxidation-reduction, which maximally reduce cell damage by regulating the adaptability of organisms to oxidative stress and hypoxia [8]. In this study, the Ad5/F35-APE1 adenoviral siRNA vector, which was constructed in our laboratory, was used to enhance the killing effect of HpD-PDT on A549 cells in vitro and in vivo, which was tested by 3-(4,5-dimethylthiazol-2-yl)-2,5-diphenyltetrazolium bromide (MTT) assay, flow cytometry, enzyme linked immunosorbent assay (ELISA) and western blot, to provide a theoretical reference for the future treatment of targeted-APE1 combined with HpD-PDT in nonsmall cell lung cancer.

## 2. Materials and Methods 

### 2.1. Cell Line, Adenovirus Vector, Instrument, and Reagents

The A549 human lung adenocarcinoma cell line (ATCC CCL-185) was generously provided by the Cancer Biotherapy Center in XinQiao Hospital of the Third Military Medical University, and it was subcultured using conventional culture conditions. The Ad5/F35-APE1 siRNA adenovirus vector was constructed and purified by Dr. Debing Xiang in our laboratory. A 630 PDT laser therapeutic apparatus with an adjustable output power of 0–2000 mW was produced by DIOMED Limited (Germany). HpD was purchased from Chongqing Municipality Huading Modern Biotechnology Limited. MTT and 2′,7′-dichlorofluorescin diacetate (DCFH-DA) were purchased from Sigma Chemical Co. (USA). The ELISA kits were purchased from NeoBioscience Co. (China).

### 2.2. MTT Assay for Analyzing the Inhibitory Effect of the Ad5/F35-APE1 siRNA Recombinant Adenovirus in Combination with HpD-PDT on the Proliferation of A549 Cells

A549 cells (1 × 10^5^/mL) were seeded in 96-well plates (100 *μ*L/well) and divided into the control, HpD-PDT, and Ad5/F35-APE1 siRNA recombinant adenovirus in combination with HpD-PDT groups. A total of 1 mg/mL HpD stock solution was diluted in RPMI 1640 complete culture medium into four concentrations: 2.5, 5, 10, and 20 *μ*g/mL. Seven doses of irradiation were used: 0.5, 1, 2, 4, 6, 8, and 10 J/cm^2^ in sextuplicate for each group. After 24 hours of culture in a 37°C, 5% CO_2_ incubator, the cells were adherent, and 200 *μ*L of the complete medium with different HpD concentrations was added. The cells were further incubated in the dark for 24 h to take up HpD fully. After various doses of irradiation, the complete medium was changed, and the cells were cultured in the dark for an additional 24 h in fresh RPMI 1640 complete medium. Next, 20 *μ*L of MTT solution (5 g/L) was added into each well, and the cells were then cultured in the dark for 4 h, and 150*μ*L of DMSO was added into each well. The plates were shaken for 10min to dissolve the purple crystals completely. The optical density (OD) at 492nm was determined for each group using a microplate reader. The mean values were obtained from triplicate experiments. The relative cell inhibition rate was calculated from the obtained OD value according to the following formula [9]: relative inhibition rate (%) = (OD value of control group − OD value of the treatment group)/OD value of the control group × 100%.

### 2.3. Flow Cytometry Analysis of Apoptosis

 A549 cells (1 × 10^5^/mL) were seeded in 96-well plates (100 *μ*L/well) and divided into the control, HpD-PDT, Ad5/F35-APE1 siRNA recombinant adenovirus, and Ad5/F35-APE1 siRNA recombinant adenovirus in combination with HpD-PDT groups. The PDT groups were irradiated with a 4 J/cm^2^ irradiation dose plus 5 *μ*g/mL HPD. The Ad5/F35-APE1 siRNA recombinant adenovirus infection dosage was 10 MOI. After 24 h of PDT treatment, the cells from each group were collected and counted, and the cell concentration was adjusted to 5 × 10^5^–1 × 10^6^/mL. A total of 1 mL of cell solution was obtained and centrifuged at 800 g for 5 min at 4°C. The supernatant was discarded, and the cells were resuspended in 4 mL of precooling PBS (4°C). After another centrifugation, 185 *μ*L of binding buffer was added to resuspend the cells. Next, 5 *μ*L of Pacific Blue annexin V and 1 *μ*L 7AAD were added. The cells were cultured while avoiding light for 20 min before flow cytometry analysis.

### 2.4. Flow Cytometry Analysis of Cellular ROS Level

Log-phase A549 cells (2 × 10^5^/mL) were seeded into 24-well plates (0.5 mL/well) and divided into the following four groups after adherence: the control, HpD-PDT, Ad5/F35-APE1 siRNA recombinant adenovirus, and Ad5/F35-APE1 siRNA recombinant adenovirus in combination with HpD-PDT groups. The PDT groups were irradiated with a 4 J/cm^2^ irradiation dose plus 5 *μ*g/mL HPD. The Ad5/F35-APE1 siRNA recombinant adenovirus infection dosage was 10 MOI. After treatment, the cells were cultured for 24 h. A total of 1.7 mg of dichlorofluorescein (DCF) was dissolved in 500 *μ*L dimethyl sulfoxide (DMSO), and 500 *μ*L of a prepared 50 mmol/L NaOH solution was later added to the DCF solution to produce a yellow-green fluorescence. After incubation while avoiding light at room temperature for 30 min, the prepared and activated DCF solution was subsequently added into a 15 mL centrifuge tube containing 6 mL PBS and placed on ice for use. The cells were washed once with PBS after treatment. The DCF probe was diluted in PBS containing 2% FCS (1 : 20), added into each well of the culture plate, and incubated with the cells for 1 h. After loading with the probe, the cells were collected in PBS and washed with twice in PBS before flow cytometry analysis.

### 2.5. Sandwich ELISA Analysis of IL-1*β* and TGF*β*1

Log-phase A549 cells (2 × 10^5^/mL) were seeded in 24-well plates (0.5 mL/well) and divided into the following four groups after adherence: the control, HpD-PDT, Ad5/F35-APE1 siRNA recombinant adenovirus, and Ad5/F35-APE1 siRNA recombinant adenovirus in combination with HpD-PDT groups. The PDT groups were irradiated with 4 J/cm^2^ of irradiation with 5 *μ*g/mL HPD. The Ad5/F35-APE1 siRNA recombinant adenovirus infection dose was 10 MOI. After treatment, the cells were cultured for 24 h. The culture medium was collected for IL-1*β* and TGF*β*1 analysis by an ELISA assay according to the manufacturer's instruction (NeoBioscience). A standard curve was plotted to calculate the concentrations of the inflammatory factors IL-1*β* and TGF*β*1.

### 2.6. Western Blot Analysis of the Time- and Dose-Effect Relationships of the Ad5/F35-APE1 siRNA Recombinant Adenovirus on APE1 Protein Expression and the Knockdown Effect of APE1 Expression Induced by HpD-PDT

A549 cells (1 × 10^5^/mL) were seeded in 6-well and 96-well plates. The cells in one of the 6-well plates were infected with a recombinant adenovirus expressing Ad5/F35-APE1 siRNA at 10 MOI. After a 4 h incubation, the old culture medium was discarded and replaced with fresh medium. The cells were collected after incubation for an additional 6, 12, 24, and 48 h. The cells in another 6-well plate were infected with 1, 2.5, 5, and 10 MOI of the Ad5/F35-APE1 siRNA recombinant adenovirus. After a 4 h incubation, the old culture medium was discarded and replaced with fresh medium. After incubation for an additional 48 h, the cells were collected, and the proteins were extracted for western blot analysis of APE1 expression as described above. The cells in the 96-well plate were divided into the control, HpD-PDT, Ad5/F35-APE1 siRNA recombinant adenovirus, and Ad5/F35-APE1 siRNA recombinant adenovirus in combination with HpD-PDT groups. The A549 cells in the adenovirus-treated groups were infected with 10 MOI of the Ad5/F35-APE1 siRNA recombinant adenovirus for 24 h. The HpD-PDT groups were irradiated with a 10 kJ/m^2^ irradiation dose plus 5 *μ*g/mL HpD. After irradiation, the cells were cultured for 24 h and were collected. The proteins were extracted for the analysis of APE1 expression as described above.

### 2.7. Statistical Analysis

Quantitative data were analyzed using SPSS 10.0 software. Significant differences among each group were determined using a *t*-test. A *P* < 0.05 was considered to be statistically significant, and a *P* < 0.01 was considered to be highly significant.

## 3. Results

### 3.1. MTT Assay for Analyzing the Inhibitory Effect of the Ad5/F35-APE1 siRNA Recombinant Adenovirus in Combination with HpD-PDT on the Proliferation of A549 Cells

**Figure 1:**
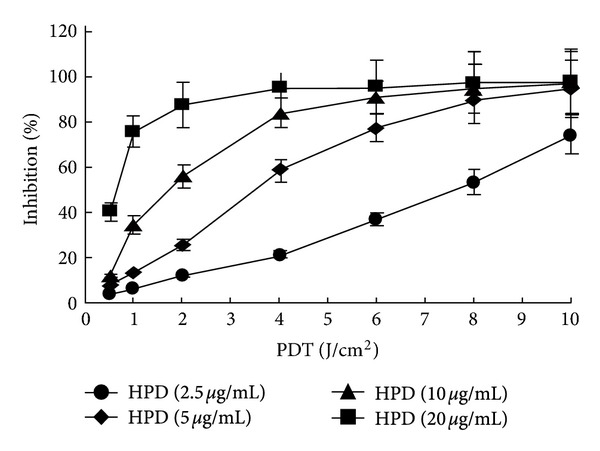
Dose-dependent inhibition of PDT on the proliferation of A549 cells. Comparisons among groups with 2.5, 5, 10, and 20 *μ*g/mL four photosensitizer concentrations and 0.5, 1, 2, 4, 6, 8, and 10 J/cm^2^ six irradiation dosages *P* < 0.01.

The results from the PDT inhibitory effect on the proliferation of A549 cells demonstrated that PDT had a significant inhibitory effect on the proliferation of A549 cells, and the inhibitory efficiency depended on the concentration of HPD and the irradiation dose. Thus, the relative A549 inhibition rate increased along with the rise in the HPD concentration and the increase in the irradiation dose. The relative suppression rate curve drastically increased at a low photodynamic dose and gradually reached a plateau (Figure 1). In addition, the results obtained with the Ad5/F35-APE1 siRNA adenovirus in combination with those of the PDT group have shown that the Ad5/F35-APE1 siRNA adenovirus could significantly enhance the PDT inhibitory effect on A549 cell proliferation. In particular, when the PDT intensity was lower than the IC_50_, the Ad5/F35-APE1 siRNA sensitization was more obvious (Figure 1).

### 3.2. Apoptosis Analysis by Flow Cytometry

**Figure 2:**
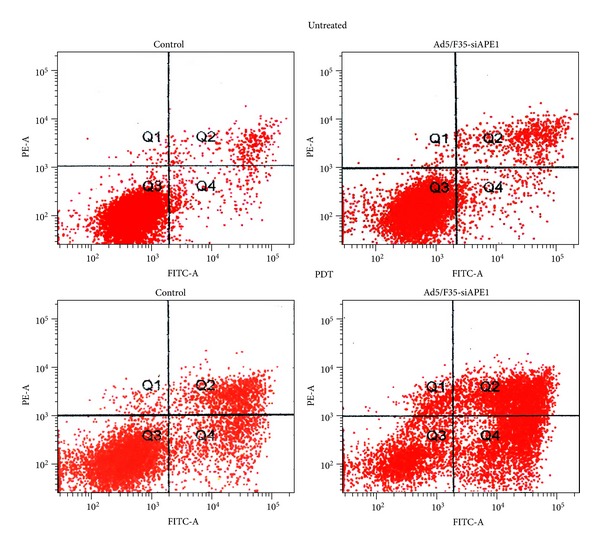
PDT induces significant apoptosis of A549 cells. Flow cytometry results of apoptotic assays after photoirradiation with or without Ad5/F35-APE1 siRNA recombinant adenovirus, untreated group as negative control. The *x* axis is for FITC staining and the *y* axis is for PE staining in all graphs represented. The cells undergoing early apoptosis are FITC positive and PE-A negative in the lower right quadrants.

Apoptosis analysis by flow cytometry demonstrated that there was no significant difference in apoptosis in the control group compared with the Ad5/F35-APE1 siRNA adenovirus group. The concentration of apoptotic cells increased after HpD-PDT treatment, but the increase was most significant in the Ad5/F35-APE1 siRNA recombinant adenovirus in combination with HpD-PDT group, which had significant differences from all of the other groups (*P* < 0.01) (Figure 2).

### 3.3. Analysis of the ROS Level

**Figure 3:**
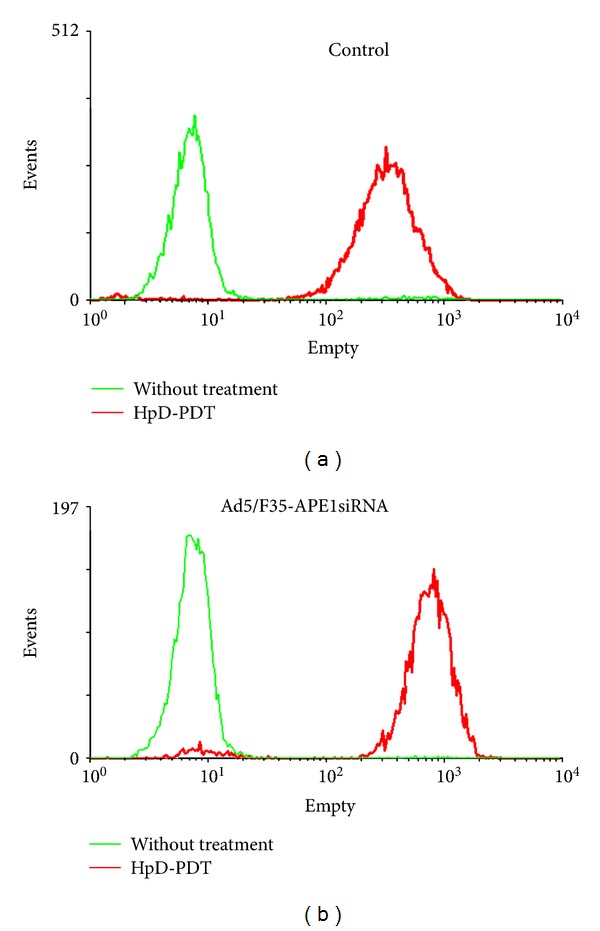
Knock down expression of APE1 promotes intracellular reactive oxygen species (ROS) production after photoirradiation. Intracellular ROS production was measured using DCFH-DA staining and assayed by flow cytometry. A549 cells received 4 kJ/m^2^ photoirradiation and were collected for ROS assay at 1, 2, and 4 hours after irradiation. ROS production at 2 hours after photoirradiation versus the HpD only control is shown in the histogram.

Compared with the control group, the intracellular ROS levels were significantly increased when A549 cells were treated with HpD-PDT and Ad5/F35-APE1 siRNA (*P* < 0.05). Additionally, Ad5/F35-APE1 siRNA in combination with PDT treatment significantly increased ROS generation, which was significantly different from all of the other groups (*P* < 0.05) (Figure 3), demonstrating that a large number of ROS were generated from photosensitizers in the target cells after stimulation with an adequate PDT wavelength.

### 3.4. ELISA Analysis of the IL-1*β* and TGF*β*1 Expression

**Figure 4:**
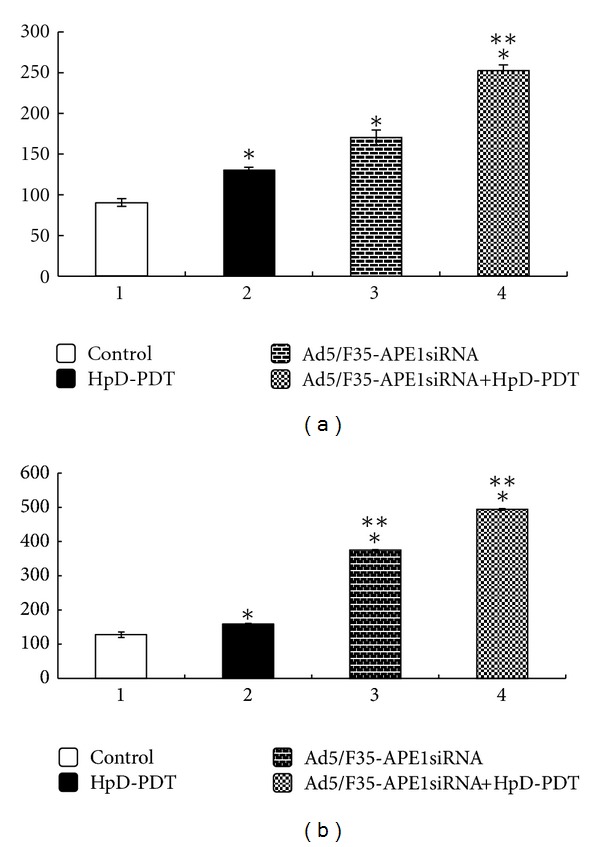
HpD-PDT-induced upregulation of IL-1*β* and TGF*β*1 expression (pg/mL). (a) IL-1*β* expression, (b) TGF*β*1 expression *P < 0.05 versus control group. **P < 0.05 versus HpD-PDT group.

Compared with the control group, all of the rest of the groups had significantly elevated IL-1*β* and TGF*β*1 protein levels in their cell culture supernatants, indicating that there were high levels of IL-1*β* and TGF*β*1 protein expression in cells after PDT irradiation. Compared with the HpD-PDT group, this increase was not significant in the Ad5/F35-APE1 siRNA group (*P* > 0.05); however, this increase was significantly higher in the Ad5/F35-APE1 siRNA in combination with HpD-PDT group (*P* < 0.05), indicating that the Ad5/F35-APE1 siRNA adenovirus could significantly induce the IL-1*β* and TGF*β*1 protein levels in the cell culture medium and could enhance the PDT killing effect on A549 cells (Figures 4(a) and 4(b)).

### 3.5. Analysis of the Time- and Dose-Effect Relationships of Ad5/F35-APE1 siRNA on APE1 Protein Expression

**Figure 5:**
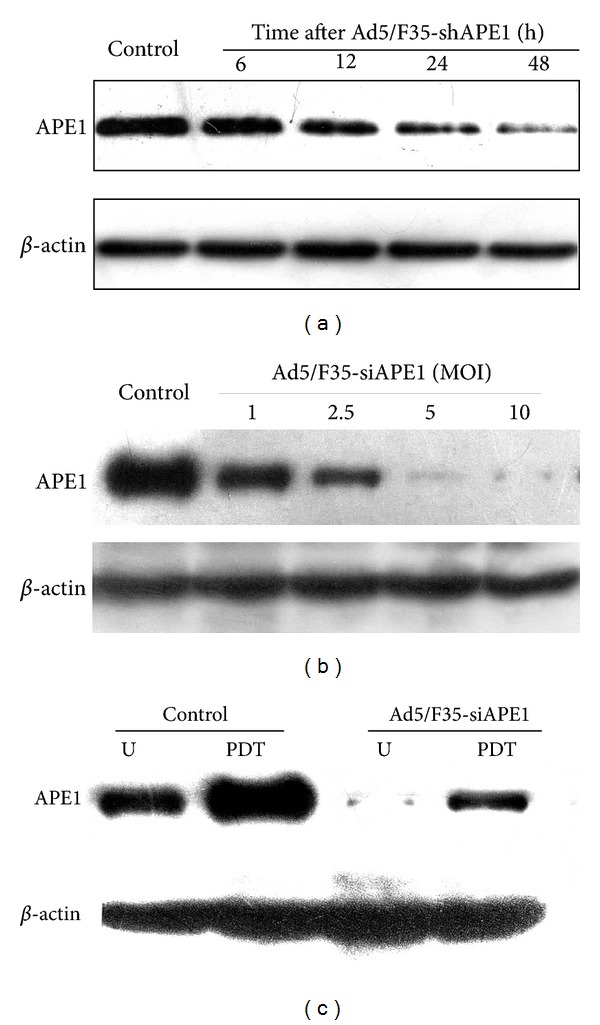
Expression of APE1 in A549 cells was knocked down by Ad5/F35-APE1siRNA in a time- and dose-dependent manner via Western blot assay. (a) Time-effect relationship of APE1 protein expression knocked down by Ad5/F35-APE1 siRNA. (b) Dose-effect relationship of the APE1 protein expression knocked down by Ad5/F35-APE1 siRNA. (c) Efficient knock down effect by Ad5/F35-APE1 siRNA on PDT-induced APE1 upregulation.

Ad5/F35-APE1 siRNA can effectively knockdown APE1 protein expression. We found that as the Ad5/F35-APE1 siRNA infection duration increased, there was an increasingly lower level of APE1 protein expression. Forty-eight hours after infection, the knockdown efficiency was the most significant (Figure 5(a)). Western blot analysis of the dose-effect relationship between the influence of the Ad5/F35-APE1 siRNA and APE1 protein expression demonstrated that the inhibitory effect on APE1 expression was most significant with an infection dose of 10 MOI of Ad5/F35-APE1 siRNA (Figure 5(b)). HpD-PDT could induce the enhancement of APE1 expression, and Ad5/F35-APE1 siRNA could efficiently knock down the upregulation of APE1 expression induced by PDT compared with the control group (Figure 5(c)).

## 4. Discussion

PDT is a type of treatment strategy that applies a minimally invasive and nonthermogenic method to induce local tissue necrosis. Compared to conventional treatments, the greatest advantage of PDT is that it selectively kills tumor cells and cause less damage to normal tissues, maximally preserving the structure and function of normal tissues and organs. This procedure may be applied in combination with surgery, radiotherapy, and chemotherapy and has promising clinical applications [3]. However, tumor cells have a capacity for self-repairing oxidative and DNA damage during treatment; thus, many PDT treatment issues remain, including limited depth of penetration, tolerance or resistance to treatment, a short effective time, and tumor recurrence. APE1 can be expressed by lung cancer cells and regulated by Hsp27, and it also induces VEGF expression [10, 11]. APE1 is an important gene that protects cells from hypoxia- and oxidative stress-induced apoptosis [12]. Through its dual functions of DNA damage repair and oxidation-reduction, APE1 can protect tumor cells from PDT-induced apoptosis, thereby affecting the therapeutic effect of PDT in the treatment of lung cancer. Our research indicates that the oxidative stress and hypoxia induced by PDT treatment can enhance APE1 expression in lung cancer cells. Therefore, understanding the function of the APE1 gene during the therapeutic response to PDT treatment in lung cancer and selecting more effective ways to inhibit APE1 expression will be expected to improve effectively the sensitivity of lung cancer cells to PDT treatment.

Many studies have shown that the downregulation of APE1 protein expression can increase the sensitivity of tumor therapies. RNA interference (RNAi) is a common gene-silencing phenomenon after transcription in eukaryotes and serves as a protective mechanism for organisms during the evolution process to fight against or inhibit viral and exogenous parasitic genes. Short-interfering-RNA- (siRNA-) induced RNAi has become a popular tool for investigating the function of specific candidate genes [13]. Research involving the RNAi mechanism has indicated that siRNAs containing only 21–25 nucleotides can specifically induce the gene silencing of candidate genes after transcription. This finding suggests that chemically synthesized siRNAs have the potential to be used as novel molecular biological tools for the specific inhibition of gene expression in mammalian cells and serves as a new technique for future cancer therapy [13–17]. Wang et al. [18] transfected a human osteosarcoma (HOS) cell line with a synthesized APE1-specific siRNA effectively to enhance the sensitivity of HOS to a DNA damaging drug. By applying antisense nucleic acid technology to specifically suppress APE1, the cell sensitivity to alkylating and oxidative drugs is increased [19, 20]. Recently, McNeill and Wilson [21] inhibited APE1 expression using a dominant-negative mutant and found that the cytotoxic effect of typical DNA-damaging drugs was significantly increased in CHO cells with up to a 5.4-fold more sensitivity to methyl methanesulfonate (MMS). Yang et al. [22] discovered that APE1 siRNA could significantly enhance the sensitivity of multiple myeloma to melphalan chemotherapy. Therefore, we used the APE1 gene as a target and blocked its transcription using Ad5/F35 recombinant adenovirus-mediated APE1 siRNA. We found that Ad5/F35-APE1 siRNA in combination with PDT could promote DNA damage in the early phase, influence the DNA repair function in the late phase, and downregulate transcription factors, such as HIF-1, to reduce VEGF protein expression and promote PDT-induced oxidative damage. In addition, our results indicate that overall, the Ad5/F35-APE1 siRNA recombinant adenovirus effectively inhibited the DNA damage repair function of APE1 in A549 cells treated with PDT, enhanced the PDT inhibitory effect on the proliferation of these cells, increased intracellular ROS expression to further influence the release of such inflammatory factors as IL-1*β* and TGF-*β*, promoted apoptosis, and increased the lung cancer sensitivity to PDT treatment. Our results suggest that gene therapy consisting of the Ad5/F35-APE1 siRNA recombinant adenovirus in combination with PDT provides a new approach for tumor therapy. Along with the development of molecular bioengineering technology, photosensitizer improvement, and the progress of PDT instrument development, we believe that the therapeutic effects of PDT in lung cancer treatment may be further improved in the near future.
